# Oral Administration of Astrocyte-Targeted Natural Antioxidants Suppress NOX4-Driven Neuroinflammation and Restore Hippocampal Neurogenesis in MPTP-Induced Parkinson’s Disease Mouse Model

**DOI:** 10.3390/nu18010055

**Published:** 2025-12-23

**Authors:** Miri Jo, Chae-Young Kim, Kayoung Ko, Seohee Choi, Jinhye Kim, Kyuhee Park, Isaac Jinwon Yi, Sang-Seop Nahm, Kiyoung Kim, Woosuk Kim, Sun-Shin Yi

**Affiliations:** 1 BK21 Four Program, Department of Medical Sciences, Soonchunhyang University, Asan 31538, Republic of Korea; jml2213@naver.com (M.J.); gurqls0404@naver.com (K.K.); suhhee1021@naver.com (S.C.); kiyoung2@sch.ac.kr (K.K.); 2 Department of Anatomy, College of Veterinary Medicine, Konkuk University, Seoul 05029, Republic of Korea; kcybada@gmail.com (C.-Y.K.); snahm@konkuk.ac.kr (S.-S.N.); 3 Central Lab, iCONNECTOME Co., Ltd., Suwon 16229, Republic of Korea; adjh08@iconnectome.com; 4 Gyeonggi Business & Science Accelerator, Suwon 16229, Republic of Korea; qheepark@gbsa.or.kr; 5 Neuroscience and Behavioral Biology Program (NBB), College of Arts and Sciences, Emory University, Atlanta, GA 30322, USA; jinwon.yi@emory.edu

**Keywords:** Parkinson’s disease, astrocyte, NOX4, neuroinflammation, redox signaling, natural antioxidants, saffron-derived antioxidant (SDA), *Passiflora incarnata* L. (PI), oral administration, neurogenesis, 1-methyl-4-phenyl-1,2,3,6-tetrahydropyridine (MPTP)

## Abstract

**Background/Objectives**: Astrocytic redox-inflammatory signaling has been implicated in Parkinson’s disease (PD) pathology and may constrain hippocampal neurogenesis. We previously identified an astrocytic NOX4–MPO–OPN axis associated with impaired neurogenic capacity. Here, we tested whether a saffron-derived antioxidant (SDA; *Crocus sativus* extract) and *Passiflora incarnata* L. extract (PI) modulate this pathway in an MPTP-induced PD mouse model. **Methods**: Male C57BL/6J mice were randomized to Sham, MPTP, and treatment groups (*n* = 9/group for behavior; *n* = 4–5/group for histology/immunoblotting). SDA or PI (50 mg/kg/day, oral, 5 weeks) was administered, with resveratrol as a positive control. Behavioral, histological, and molecular analyses were performed by investigators blinded to group allocation where feasible. **Results**: SDA and PI were associated with reduced NOX4/MPO/OPN signals, mainly in GFAP-positive astrocytes, along with recovery of neurogenesis markers (Ki67, DCX, BrdU/NeuN) and synaptic markers (PSD95, synaptophysin), and improved motor performance. Mitochondrial and oxidative injury markers (TIM23, TOM20, OXPHOS subunits; 4-HNE) and apoptotic markers (Bax, cleaved caspase-3, Bcl-2) also shifted toward Sham levels. Given previous reports of Passiflora extracts’ sedative effects, we note that metabolic measures (body weight, food intake, and water intake) were similar across groups; however, specific tests for sedation or arousal were not conducted. **Conclusions**: These findings offer preclinical evidence that SDA and PI modulate redox-inflammatory and mitochondrial stress signatures and are associated with neurogenic, synaptic, and behavioral improvements in an acute MPTP model. Further validation in chronic/genetic PD models and pharmacokinetic/brain exposure studies will be necessary to confirm their translational potential.

## 1. Introduction

Parkinson’s disease (PD) is not only characterized by the loss of dopaminergic neurons but is increasingly recognized as a disorder influenced by non-neuronal cells, especially glia [1,2,3,4]. Among these, astrocytes play a central role in neuroimmune interactions, and their dysregulation may accelerate neuronal death through redox-sensitive inflammatory mechanisms [5,6,7]. PD involves the progressive degeneration of dopaminergic neurons in the substantia nigra pars compacta (SNc), the buildup of misfolded α-synuclein in Lewy bodies, and motor dysfunction along with cognitive decline [4,8,9,10,11]. Importantly, recent findings suggest that neuroinflammation and oxidative stress in glial cells—particularly astrocytes—may occur before or exacerbate these key pathologies, supporting the development of glial-targeted therapies [12,13,14]. Beyond the degeneration of the dopaminergic system, neurogenesis in the hippocampus and subventricular zone (SVZ) is significantly impaired in PD patients [15,16,17]. Adult neurogenesis in the dentate gyrus (DG) is vital for cognitive flexibility, emotional regulation, and recovery after neuroinflammatory insults. Its decline has been linked to disease progression and resistance to treatment [18,19,20]. Growing evidence indicates that dysfunction in astrocytic redox regulation may be a significant obstacle to regenerative processes within the neurogenic niches of the PD brain [21,22,23,24].

Oxidative stress is increasingly recognized as a key driver of neuroinflammation in the PD [25,26]. Specifically, the enzyme NADPH oxidase 4 (NOX4)—mainly expressed in astrocytes—produces reactive oxygen species (ROS) that trigger cascades of neurotoxicity, including lipid peroxidation, cytokine release, and ferroptosis cell death [27,28]. Our previous research identified NOX4-driven upregulation of myeloperoxidase (MPO) and osteopontin (OPN) in hippocampal astrocytes as a crucial mechanism of neurodegeneration and suppression of neurogenesis in both PD and Alzheimer’s models [27,29]. Therefore, targeting this NOX4–MPO–OPN axis offers a novel astrocyte-centered approach to reduce neuroinflammation and promote hippocampal repair.

Current pharmacological treatments for PD, including dopamine-replacement strategies like L-DOPA, provide symptomatic relief but do not modify the disease course or promote neuroregenesis [30]. Additionally, long-term use of these agents often leads to adverse effects such as dyskinesia and treatment tolerance [31,32]. These challenges highlight the need for adjunct therapies that not only reduce neuroinflammation but also restore the neurogenic and synaptic structures of the diseased brain. In this context, naturally derived antioxidants with glial-targeted redox activity are promising therapeutic candidates. Saffron-derived antioxidant (SDA; *Crocus sativus* extract) and *Passiflora incarnata* L. (PI) are phytochemicals with well-documented antioxidant, anti-inflammatory, and neurotrophic properties and established safety profiles for human consumption.

Although traditionally explored in stress or sleep modulation, their mechanisms of action have not been thoroughly studied in the context of neuroinflammation-linked neurodegeneration. Our previous studies demonstrated that SDA modulates BDNF and HPA axis activity [33], while PI promotes hippocampal neurogenesis and enhances cognition in sleep-deprivation models [34]. However, their relevance to glial redox regulation in PD has not been explored. In this study, we examined whether the oral administration of these two natural compounds—SDA and PI—can inhibit NOX4-driven neuroinflammation and restore hippocampal neurogenesis in an MPTP-induced mouse model of PD. We hypothesized that these safe, plant-derived extracts would decrease astrocytic NOX4-MPO-OPN signaling, restore synaptic markers, and promote neuronal differentiation, ultimately enhancing motor and cognitive functions. This approach supports the development of glial-targeted redox modulators as accessible, long-term nutritional strategies for managing PD progression.

## 2. Materials and Methods

### 2.1. Test Substance

Resveratrol (RES; Activ’Inside, Beychac-et-Caillau, France), which was employed as a positive control in the present study due to its previously reported neuroprotective and antioxidant actions in MPTP-induced PD models [35,36,37,38,39]. RES, SDA (Pharmactive Biotech Products S.L., Madrid, Spain), and PI were accurately weighed and transferred into sterile 15 mL centrifuge tubes. The PI extract used in this study was prepared from leaves and fruits and corresponded to the same standardized extract characterized in our previous work [34]. Each substance was freshly suspended in distilled water (D.W.; vehicle) and vortexed immediately prior to administration. The final dosing volume was adjusted to deliver an oral dose of 50 mg/kg/day for RES, SDA, and PI, consistent with effective dosing regimens reported in prior in vivo studies. Based on previous MPTP studies showing that 50 mg/kg/day RES maintained striatal dopaminergic markers [35,36,37], and on in vivo work using similar oral doses of saffron extracts or saffron-derived compounds [40,41,42], we selected 50 mg/kg/day as a pharmacologically effective yet nutritionally relevant dose for SDA. For PI, prior mouse studies in sleep-related models reported efficacy at 10 and 50 mg/kg [34], we therefore adopted 50 mg/kg/day to match the SDA and RES regimens. These considerations collectively supported the use of a unified oral dose of 50 mg/kg/day for RES, SDA, and PI in the present study.

### 2.2. Animals and Experimental Design

Male C57BL/6J mice (8 weeks old, 21 ± 1 g; Jackson Laboratory, Bar Harbor, ME, USA) were housed under controlled conditions (22 ± 2 °C, 60% humidity, 12 h light/dark cycle) with ad libitum access to chow (2018S; Harlan, Indianapolis, IN, USA) and D.W. Body weight, food intake, and water consumption were recorded daily for five weeks. All procedures were approved by the Institutional Animal Care and Use Committee (IACUC) of Soonchunhyang University (Approval No. SCH24-0015).

Mice (*n* = 45) were randomly assigned to five experimental groups (*n* = 9 per group), with four mice used for histology and five for protein analysis. Parkinsonism was induced in Groups 2–5 by daily intraperitoneal (i.p.) injection of MPTP hydrochloride (30 mg/kg/day; Selleckchem, TX, USA) for four weeks. Group 1 (Sham) received saline (0.9% NaCl). Natural extract treatments were orally administered (p.o.) once daily for five weeks: SDA (50 mg/kg/day), PI (50 mg/kg/day), RES (50 mg/kg/day) as a positive control. Groups 1 and 2 received vehicle (D.W.) (Supplementary Figure S1).

Experimental groups were as follows:

1. Group 1 (Sham): Saline + D.W. (vehicle);
2. Group 2 (MPTP): MPTP (30 mg/kg) + D.W.;
3. Group 3 (MPTP + RES): MPTP (30 mg/kg) + RES (50 mg/kg);
4. Group 4 (MPTP + SDA): MPTP (30 mg/kg) + SDA (50 mg/kg);
5. Group 5 (MPTP + PI): MPTP (30 mg/kg) + PI (50 mg/kg).

Proliferating cells were labeled using intraperitoneal injections of 5-bromo-2’-deoxyuridine (BrdU; 50 mg/kg; Sigma-Aldrich, St. Louis, MO, USA) twice daily for three consecutive days at the study onset.

### 2.3. Behavioral Assessments

Grip strength test: Forelimb strength was measured using a grip strength meter (Jeung Do Bio & Plant Co., Ltd., Seoul, Republic of Korea). Each mouse grasped a horizontal bar attached to a force gauge, and the peak force during gentle tail traction was recorded. Measurements were performed three times with 5 min intervals, and the mean force was calculated.

Rotarod test: Motor coordination was assessed using a rotating rod (CASSCALE KOREA, Seoul, Republic of Korea) set at a constant speed of 20 rpm. Latency to fall was recorded (maximum trial duration: 300 s). Each mouse underwent three trials separated by 5 min rest periods, and the average latency was calculated.

Wire hang test: Neuromuscular endurance was evaluated using an inverted wire cage lid suspended 50 cm above a cushioned surface. The duration until falling (maximum 500 s per trial) was recorded. Each mouse completed three trials with 10 min recovery intervals, and the average duration was computed.

### 2.4. Histological Analysis

At the completion of the study, animals were anesthetized (urethane, 20%, 10 mL/kg, i.p.; Daejung Chemicals, Gyeonggi-do, Republic of Korea) and then transcardially perfused with saline, followed by 4% paraformaldehyde (PFA). Brains were extracted, post-fixed overnight in 4% PFA, and paraffin-embedded. Coronal brain sections (4 µm) were prepared according to standard stereotaxic coordinates for hippocampus (−1.46 to −2.06 mm) and substantia nigra (−2.80 to −3.28 mm).

Nissl staining: Sections were stained with 1% cresyl violet (Daejung Chemicals) for 30 min, sequentially dehydrated, cleared, and mounted using Eukitt^®^ Quick-hardening mounting medium (Sigma-Aldrich).

Immunohistochemistry (IHC): Sections underwent antigen retrieval, hydrogen peroxide treatment (0.3%), and blocking (CAS-Block™; Thermo Fisher Scientific, Waltham, MA, USA). Primary antibodies targeting α-synuclein (1:500, Santa Cruz Biotechnology, Dallas, TX, USA), Doublecortin (DCX; 1:200, Santa Cruz Biotechnology), Ki67 (1:500, Abcam, Cambridge, UK), and tyrosine hydroxylase (TH; 1:500, Abcam) were incubated overnight (4 °C). Secondary biotinylated antibodies (Vector Laboratories, Burlingame, CA, USA) were incubated for 2 h at room temperature. Visualization utilized an avidin-biotin-peroxidase complex kit (Vectastain Elite ABC Kit; Vector Laboratories) and DAB (Sigma-Aldrich). Sections were dehydrated and mounted as described above. Quantification of immunopositive cells was performed using ImageJ software (v1.53g, NIH, Bethesda, MD, USA). Ki67-positive cells were manually counted within the subgranular zone (SGZ), and DCX-positive cells were counted in the granule cell layer (GCL)–SGZ of the DG. The mean number of positive cells per section was calculated and expressed as mean ± SEM.

Immunofluorescence (IF): Sections underwent citrate buffer antigen retrieval and blocking before incubation with primary antibodies (overnight, 4 °C): BrdU (1:500, Cell Signaling, Boston, MA, USA), Glial Fibrillary Acidic Protein (GFAP; 1:300, Millipore, Burlington, MA, USA), MPO (1:100, R&D Systems, Minneapolis, MN, USA), Neuronal Nuclei (NeuN; 1:500, Abcam), NOX4 (1:100, Santa Cruz), OPN (1:500, R&D Systems), Postsynaptic Density Protein-95 (PSD95; 1:500, Invitrogen, Waltham, MA, USA), and Synaptophysin (1:100, Novus, Centennial, CO, USA). Appropriate fluorescent secondary antibodies (Invitrogen, Jackson ImmunoResearch, West Grove, PA, USA) were applied for 2 h at room temperature, with nuclei counterstained using DAPI (Vector Laboratories). BrdU labeling required DNA denaturation with formamide and hydrochloric acid before antibody incubation. The fluorescence images were acquired under identical exposure and gain settings. The total fluorescence intensity of each analyzed hippocampal field was quantified using ImageJ software (NIH) and divided by the corresponding field area (mm^2^), yielding intensity per mm^2^. This normalization accounted for differences in imaging area across samples. BrdU-positive cells were identified based on nuclear BrdU immunoreactivity within the GCL–SGZ of the DG. Cells double-labeled with BrdU and NeuN were defined as newly generated neurons (BrdU^+^/NeuN^+^), whereas cells double-labeled with BrdU and GFAP were defined as astrocytic lineage cells (BrdU^+^/GFAP^+^). The proportion of each cell type was calculated as the percentage of double-positive cells among total BrdU-positive cells.

### 2.5. Western Blotting

Hippocampal proteins were extracted in RIPA buffer containing phosphatase inhibitors. Protein concentrations were measured, and equal amounts were subjected to SDS-PAGE and transferred onto PVDF membranes (Cytiva, Marlborough, MA, USA). After blocking with TBST containing 5% BSA, membranes were incubated overnight (4 °C) with primary antibodies: 4-Hydroxynonenal (4-HNE; 1:1000, Bioss, Beijing, China), Bcl-2-Associated X Protein (BAX; 1:1000, Cell signaling Technology), B-Cell Lymphoma 2 (Bcl-2; 1:1000, Cell signaling Technology), cleaved caspase-3 (CC3; 1:500, Cell Signaling Technology), DCX (1:1000, Abcam), GFAP (1:1000, Merck Millipore, Burlington, MA, USA), Ionized calcium binding adaptor molecule 1 (Iba-1; 1:200, Wako, Osaka, Japan), MPO (1:500, R&D systems), OPN (1:100, R&D Systems), NeuN (1:500, Abcam), NOX4 (1:100, Novus, Centennial, CO, USA), PSD95 (1:1000, Invitrogen, Waltham, MA, USA), Synaptophysin (1:20,000, Novus), TIM23 (1:1000, Proteintech, Chicago, IL, USA), Tom20 (1:100, Santa Cruz Biotechnology), Total OXPHOS (1:250, Abcam) and Glyceraldehyde-3-Phosphate Dehydrogenase (GAPDH; 1:10,000, Cell Signaling). After washing, membranes were incubated with horseradish peroxidase (HRP)-conjugated secondary antibodies (Vector Laboratories, Cell Signaling) for 2 h, and immunoreactive bands were visualized using enhanced chemiluminescence reagents (BS ECL Plus Kit; Biosesang, Yongin-si, Gyeonggi-do, Republic of Korea). For Western blot (WB) analyses, the protein expression levels of each target were normalized to GAPDH and expressed as fold changes relative to the Sham group (set as 1.0). Fold change values were then compared across experimental groups. Differences considered statistically significant at *p* < 0.05.

### 2.6. Statistical Analysis

High-resolution IHC imaging was performed using the MoticEasyScan One Slide Scanner (Motic, Xiamen, China). IF images were acquired using an Olympus BX53F microscope (Olympus Corporation, Tokyo, Japan). Statistical analyses were conducted using GraphPad Prism 10.4 software (GraphPad Software, Boston, MA, USA). Group comparisons were performed using one-way ANOVA followed by Dunnett’s post hoc test. Data are expressed as mean ± standard error of the mean (SEM). Investigators performing behavioral testing, histological counts, and densitometry were blinded to group allocation.

## 3. Results

### 3.1. SDA and PI Preserve Dopaminergic Neurons, Reduce α-Synuclein Accumulation, and Improve Motor Performance in MPTP-Induced PD Mice

**Figure 1:**
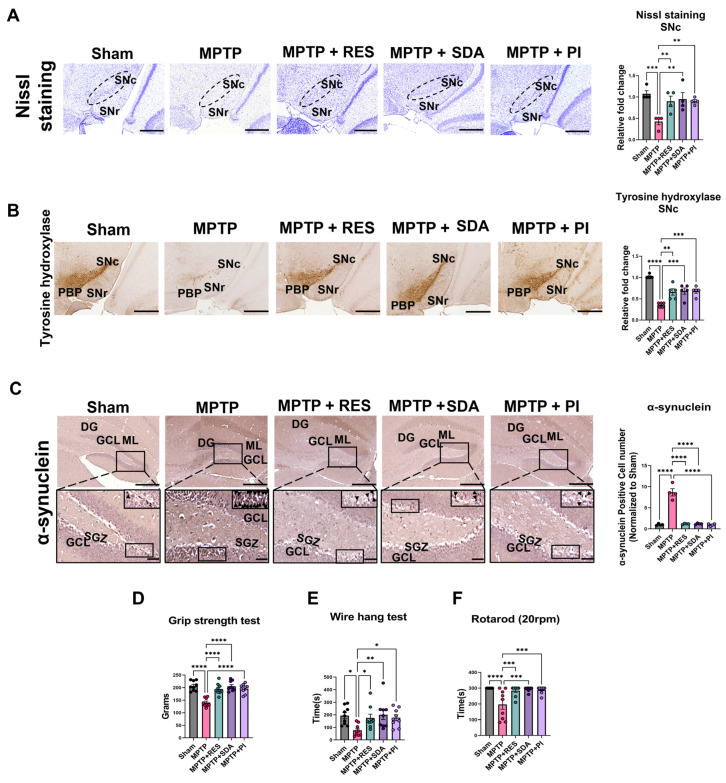
SDA and PI protect dopaminergic neurons, reduce α-synuclein accumulation, and improve motor function in MPTP-treated mice. (**A**) Representative Nissl-stained sections of the SNc showing neuronal density loss in MPTP-treated mice, with protective effects observed following SDA and PI treatment. Quantification of Nissl-positive cells is expressed as a fold change relative to the Sham group. (**B**) TH IHC in the SNc indicates dopaminergic neuron survival. Quantified TH-positive staining is shown as a fold change versus Sham. (**C**) Immunohistochemical detection of α-synuclein in the DG, with boxed regions magnified to highlight α-synuclein-positive cells (arrowheads) in the SGZ and GCL. (**D**–**F**) Behavioral tests assessing motor performance: (**D**) Forelimb grip strength (grams), (**E**) Rotarod latency at a constant 20 rpm (seconds), (**F**) Wire hang endurance (seconds). Each test was conducted in triplicate with rest intervals of 5–10 min, and mean values were used for analysis. Scale bars: 200 µm (**A**–**C** upper panels); 100 µm (**C** lower panels). Data represent mean ± SEM (*n* = 4 for histology, *n* = 9 for behavioral tests). *, *p* < 0.05; **, *p* < 0.01; ***, *p* < 0.005; ****, *p* < 0.0001. DG: dentate gyrus; GCL: granule cell layer; ML: molecular layer; PBP: parabrachial pigmented nucleus of the VTA; SGZ: subgranular zone; SNc: substantia nigra pars compacta; SNr: substantia nigra pars reticulata.

Nissl staining and TH IHC revealed marked neuronal loss and dopaminergic degeneration in the SNc of MPTP-treated mice compared with Sham controls. Quantitative analysis confirmed that treatment with RES, SDA, or PI significantly preserved neuronal density and TH-positive dopaminergic neurons in the SNc (Figure 1A,B). In addition, MPTP-induced accumulation of α-synuclein was prominently observed in the GCL–SGZ of the DG, and this pathological aggregation was substantially reduced by administration of all three compounds (Figure 1C). Consistent with these histological findings, behavioral assessments demonstrated that MPTP-treated mice exhibited significant impairments in grip strength, rotarod performance, and wire-hang endurance, which were markedly ameliorated following treatment. One-way ANOVA revealed significant group differences in grip strength (F(4,40) = 17.93, *p* < 0.0001), rotarod latency (F(4,40) = 8.034, *p* < 0.0001), and wire-hang duration (F(4,40) = 3.428, *p* = 0.0168). Among the treatment groups, SDA produced the most pronounced improvements in grip strength and wire-hang performance, suggesting enhanced preservation of neuromuscular function (Figure 1D–F). Importantly, no significant differences in body weight, food intake, or water consumption were detected among groups, indicating that the observed behavioral improvements were not confounded by systemic metabolic factors (Supplementary Figure S2). Collectively, these results suggested that SDA and PI confer significant neuroprotection against MPTP-induced dopaminergic damage, suppress α-synuclein pathology, and restore motor function, underscoring their therapeutic potential as glial-targeted redox modulators in PD.

### 3.2. SDA and PI Restore Hippocampal Neurogenesis Impaired by MPTP

**Figure 2:**
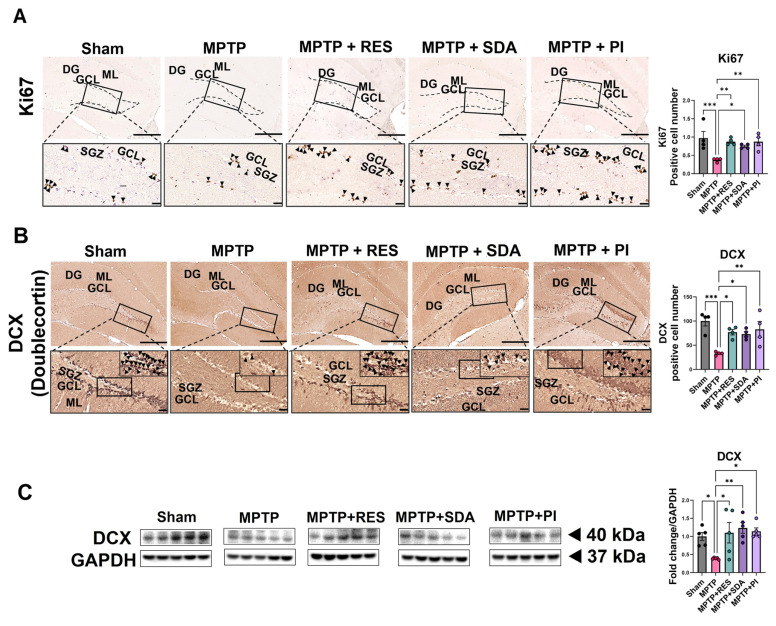
SDA and PI enhance hippocampal neurogenesis by promoting cell proliferation and neuronal development. (**A**) IHC for Ki67 in the SGZ of the DG shows dividing cells (arrowheads). Counting Ki67^+^ cells reveals significant decreases in the MPTP group, which are reversed by treatment with SDA, PI, or RES. (**B**) DCX staining highlights immature neurons within the SGZ–GCL. Boxed areas are enlarged to display representative DCX^+^ cells (arrowheads). (**C**) WB analysis of DCX protein levels in hippocampal extracts, normalized to GAPDH, supports histological evidence of restored neurogenesis. Scale bars: 200 µm (upper panels); 100 µm (lower panels). Data represent mean ± SEM (*n* = 4, 5 per group). *, *p* < 0.05; **, *p* < 0.01; ***, *p* < 0.005. DG: dentate gyrus; GCL: granule cell layer; ML: molecular layer; SGZ: subgranular zone.

Immunohistochemical analysis of the DG revealed a marked reduction in Ki67-positive proliferating cells and DCX-positive immature neurons in MPTP-treated mice, confirming that MPTP intoxication significantly suppressed hippocampal neurogenesis. Treatment with RES, SDA, or PI effectively reversed these deficits, restoring both proliferative and differentiating neuronal populations (Figure 2A–C). Among the treatment groups, PI produced the most pronounced enhancement of neurogenesis, followed by SDA and RES, suggesting distinct efficacies in promoting hippocampal cell renewal. Consistent with these histological findings, WB analysis showed a significant increase in DCX protein expression in all treatment groups compared with MPTP controls, corroborating the restoration of neurogenic activity at the molecular level (Figure 2C). Collectively, these results indicate that both SDA and PI effectively counteract MPTP-induced impairment of hippocampal neurogenesis, with PI exhibiting the strongest neurogenic potential.

### 3.3. SDA and PI Restore Synaptic Plasticity Markers in the Hippocampus

**Figure 3:**
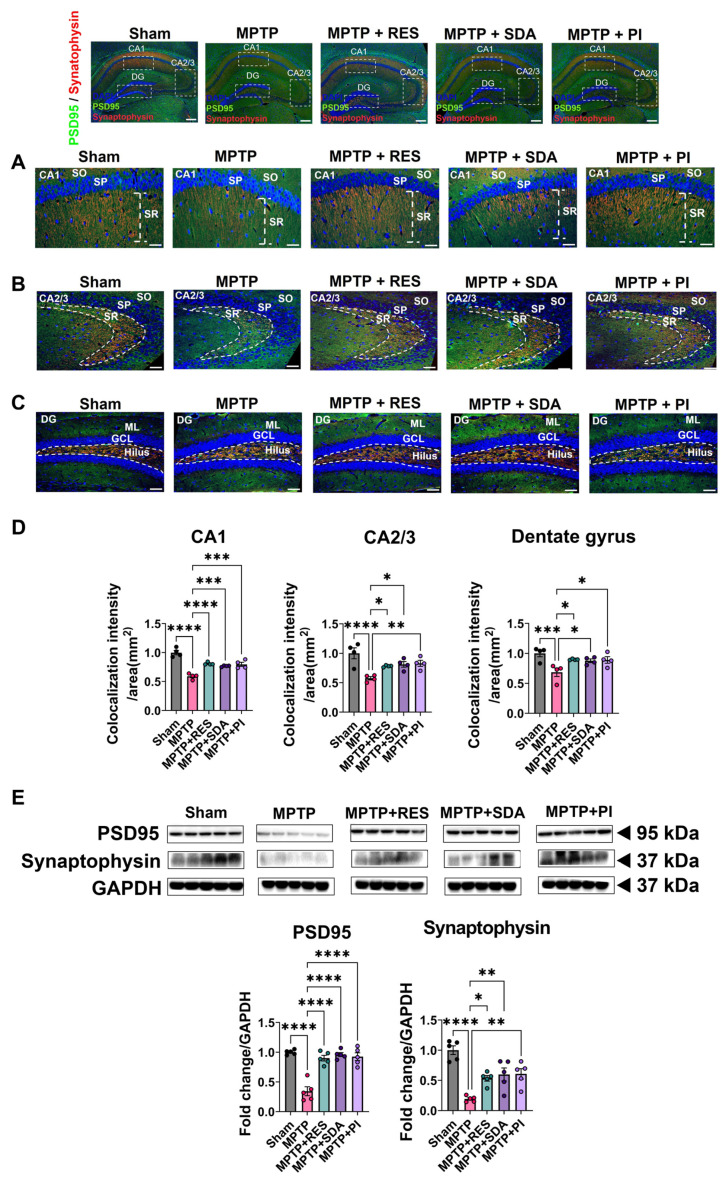
SDA and PI restore hippocampal synaptic plasticity through PSD95 and synaptophysin expression. (**A**–**D**) Representative double IF images showing PSD95 (green) and synaptophysin (red) expression in hippocampal subregions (CA1, CA2/3, and DG). RES, SDA, and PI treatments reversed MPTP-induced reductions in co-localization. Quantification of co-localized PSD95 and synaptophysin is presented as intensity per mm^2^. (**E**) WB analysis of hippocampal PSD95 and synaptophysin expression normalized to GAPDH. MPTP decreased both synaptic markers, which were significantly restored by all treatments. Scale bars: 500 µm (top), 100 µm (bottom). Data represent mean ± SEM (*n* = 4–5 per group). *, *p* < 0.05; **, *p* < 0.01; ***, *p* < 0.005; ****, *p* < 0.0001. CA1: Cornu Ammonis 1; CA2/3: Cornu Ammonis 2/3; DG: Dentate gyrus; GCL: granule cell layer; ML: molecular layer; SO: stratum oriens; SP: stratum pyramidale; SR: stratum radiatum.

To evaluate synaptic integrity, the expression of postsynaptic density protein 95 (PSD95) and synaptophysin was examined in the hippocampal CA1, CA2/3 (Cornu Ammonis Fields 1–3), and DG regions. MPTP administration markedly reduced the expression of both markers, indicating substantial disruption of synaptic architecture. Treatment with RES, SDA, or PI significantly restored PSD95 and synaptophysin expression to near-Sham levels (Figure 3A–D). Double IF analysis revealed pronounced regional recovery of PSD95 and synaptophysin in CA1, CA2/3, and DG, with increased colocalization intensity suggesting improved pre- and postsynaptic connectivity (Figure 3A–D). These findings were further corroborated by WB analysis, which confirmed the restoration of PSD95 and synaptophysin protein levels in all treatment groups (Figure 3E). Collectively, these results suggested that SDA and PI effectively reverse MPTP-induced synaptic damage and re-establish hippocampal connectivity, providing structural support for their neuroprotective efficacy.

### 3.4. SDA and PI Promote Neuronal over Astrocytic Differentiation of Newly Generated Cells

**Figure 4:**
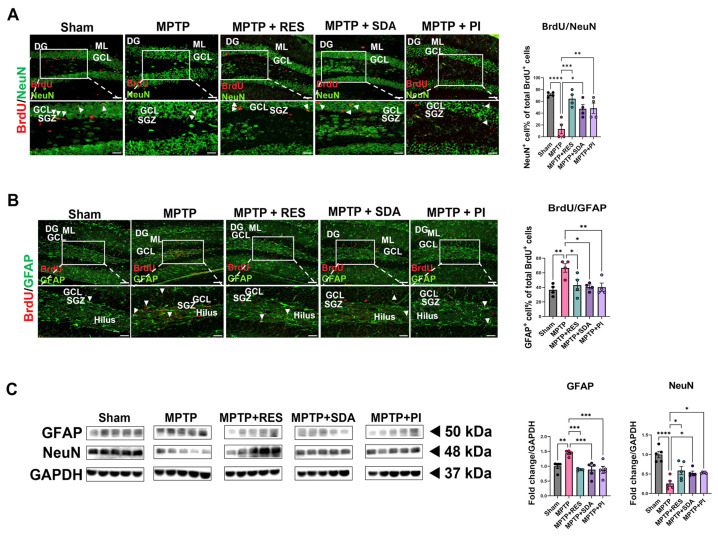
SDA and PI promote neuronal over astrocytic differentiation of newly generated cells in the DG. (**A**) Representative double IF images showing co-localization of BrdU (red) and NeuN (green) in the DG. Arrowheads indicate BrdU^+^/NeuN^+^ double-positive cells representing newly generated neurons. (**B**) Representative images of BrdU (red) and GFAP (green) co-staining showing BrdU^+^/GFAP^+^ cells, indicating astrocytic lineage commitment. (**C**) WB analysis of NeuN and GFAP protein levels in hippocampal lysates, normalized to GAPDH. Scale bars: 100 µm (upper panels), 50 µm (magnified panels). Data represent mean ± SEM (*n* = 4–5 per group). *, *p* < 0.05; **, *p* < 0.01; ***, *p* < 0.005; ****, *p* < 0.0001. DG: Dentate gyrus; GCL: granule cell layer; ML: molecular layer; SGZ: subgranular zone.

To investigate the lineage fate of newly proliferated cells, BrdU was co-stained with NeuN or GFAP. MPTP exposure favored astrocytic differentiation (more BrdU^+^/GFAP^+^ cells) and suppressed neuronal fate (fewer BrdU^+^/NeuN^+^ cells). This trend was reversed by all treatments, with SDA and PI encouraging neurogenic differentiation. WB analysis supported these results, showing increased NeuN and decreased GFAP levels in hippocampal tissue after treatment (Figure 4A–C). Quantification of BrdU^+^/NeuN^+^ and BrdU^+^/GFAP^+^ cell populations showed a significant shift toward neuronal line commitment following treatment. These IF results were further substantiated by increased NeuN and decreased GFAP protein expression on WB (Figure 4C).

### 3.5. SDA and PI Suppress NOX4, MPO, and OPN Expression in the Hippocampus

**Figure 5:**
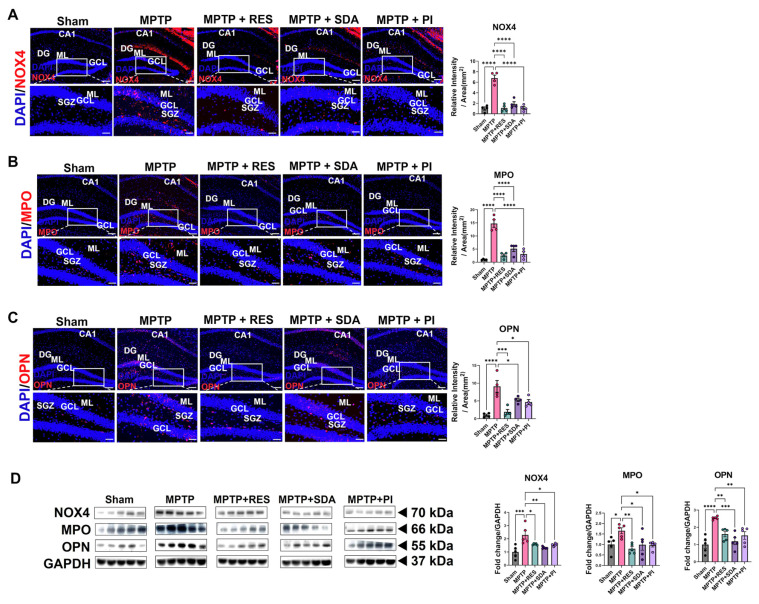
SDA and PI decrease NOX4, MPO, and OPN expression in the hippocampus. (**A**–**C**) Representative double IF images showing NOX4 (**A**), MPO (**B**), and OPN (**C**) (red) in the DG. Nuclei are counterstained with DAPI (blue). Treatment with SDA and PI significantly reduced the fluorescence intensity of all three markers compared with the MPTP group. (**D**) WB analysis of NOX4, MPO, and OPN protein levels in hippocampal lysates. Densitometric quantification, normalized to GAPDH, is expressed as fold change relative to the Sham group. Quantitative analysis of WB data showing significant upregulation of NOX4, MPO, and OPN in the MPTP group and their marked reduction following treatment with RES, SDA, or PI. Both SDA and PI exhibited efficacy comparable to that of RES in attenuating oxidative/inflammatory protein expression in the hippocampus. Scale bars: 200 µm (upper panels), 100 µm (magnified). Data represent mean ± SEM (*n* = 4–5 per group). *, *p* < 0.05; **, *p* < 0.01; ***, *p* < 0.005; ****, *p* < 0.0001. CA1: Cornu Ammonis 1; DG: Dentate gyrus; GCL: granule cell layer; ML: molecular layer; SGZ: subgranular zone.

To evaluate the modulation of oxidative-inflammatory pathways, we measured hippocampal levels of NOX4, MPO, and OPN. MPTP treatment significantly raised all three markers. These increases were strongly reduced by RES, SDA, and PI, as confirmed by IF and WB analysis (Figure 5A–D). IF imaging localized these redox markers to the DG, with markedly attenuated fluorescence intensities across all treatment groups. WB quantitatively confirmed these decreases in NOX4, MPO, and OPN (Figure 5D).

### 3.6. Cell-Type Specific Suppression of NOX4, MPO, and OPN by SDA and PI

**Figure 6:**
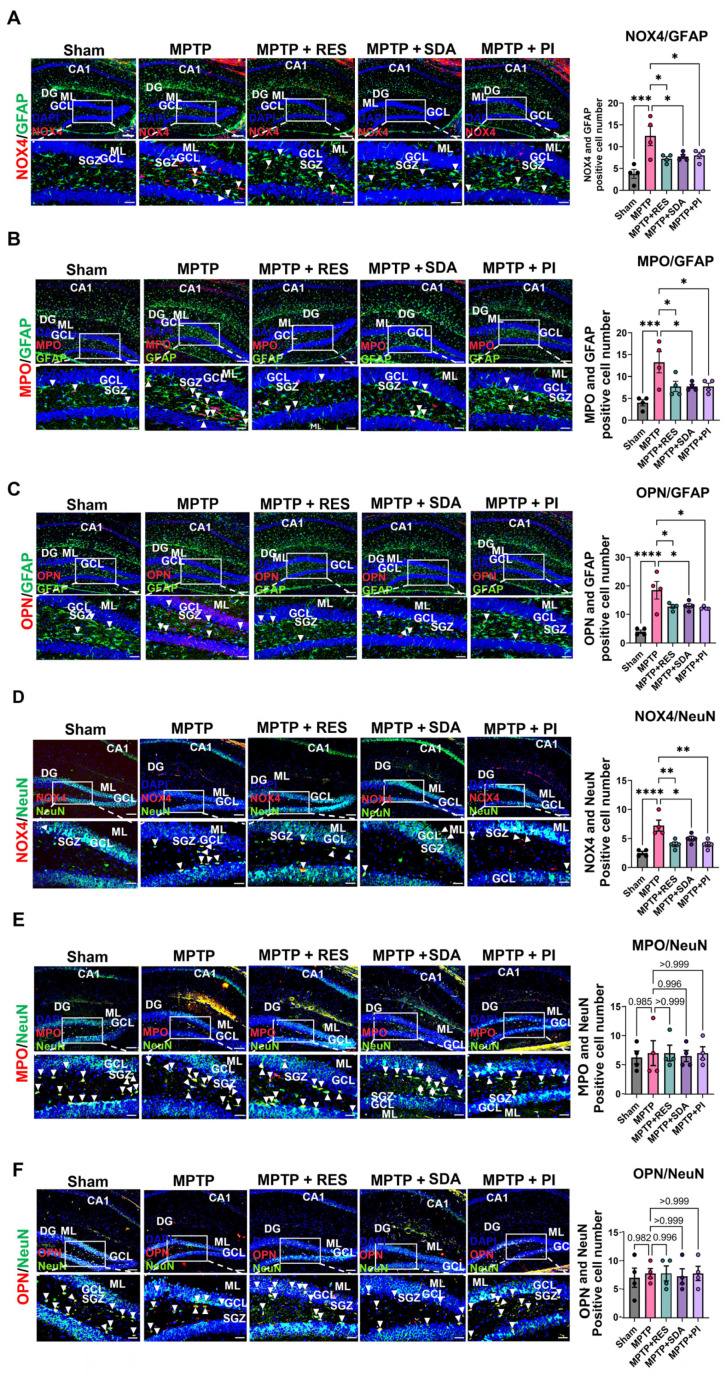
SDA and PI selectively inhibit NOX4, MPO, and OPN expression in hippocampal astrocytes and neurons. (**A**–**C**) Representative double IF images showing colocalization of NOX4 (**A**), MPO (**B**), and OPN (**C**) (red) with GFAP (green) in astrocytes of the DG. Arrowheads indicate GFAP^+^ cells co-expressing each inflammatory marker. Treatment with SDA and PI markedly reduced the number of double-positive astrocytes. (**D**–**F**) Colocalization analysis of NOX4 (**D**), MPO (**E**), and OPN (**F**) (red) with NeuN (green) in neurons. Notably, only NOX4 expression was significantly decreased in NeuN^+^ cells after treatment, while MPO and OPN expression largely remained unchanged in neurons. Quantification of double-positive colocalized cells is displayed in the accompanying bar graphs. Scale bars: 200 µm (upper panels), 100 µm (magnified). Data represent mean ± SEM (*n* = 4 per group). *, *p* < 0.05; **, *p* < 0.01; ***, *p* < 0.005; ****, *p* < 0.0001. CA1; Cornu Ammonis 1, DG; Dentate gyrus, GCL; granule cell layer, ML; molecular layer, SGZ; subgranular zone.

To clarify cell-type-specific modulation, we analyzed colocalization of NOX4, MPO, and OPN with GFAP (an astrocyte marker) or NeuN (a neuronal marker). MPTP exposure significantly increased NOX4, MPO, and OPN in GFAP^+^ astrocytes, and all treatments markedly attenuated these increases (Figure 6A–C). Additionally, NOX4 levels were higher in NeuN^+^ neurons and decreased with treatment. However, MPO and OPN levels in neurons did not change (Figure 6D–F), indicating a preferential effect on astrocytic oxidative stress pathways. Notably, NOX4 expression was significantly decreased in both GFAP^+^ astrocytes and NeuN^+^ neurons, while MPO and OPN reductions were mainly limited to astrocytic regions. Neuronal MPO and OPN levels remained unchanged across groups (Figure 6E,F), underscoring the astrocyte-specific targeting of SDA and PI.

### 3.7. Natural Extract Treatment Restores Mitochondrial Integrity and Suppresses Apoptosis in the Hippocampus Lysates of MPTP-Induced PD Mice

**Figure 7:**
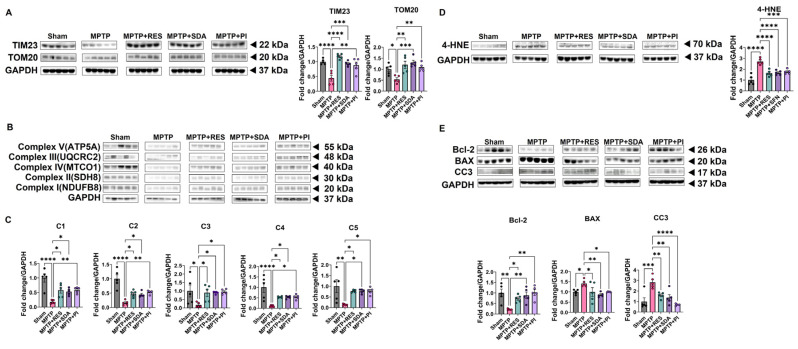
SDA and PI restore mitochondrial integrity and inhibit apoptosis in the hippocampus. (**A**) Representative WB showing hippocampal expression of mitochondrial import and translocase proteins TIM23 and TOM20. Both SDA and PI significantly restored these mitochondrial markers that were reduced in MPTP-treated mice. (**B**,**C**) WB analysis of OXPHOS complex subunits I (NDUFB8), II (SDH8), III (UQCRC2), IV (MTCO1), and V (ATP5A). Quantitative densitometry demonstrates that MPTP treatment markedly suppressed each complex, whereas SDA and PI restored their levels toward those of the Sham group. (**D**) WB and densitometric analysis of 4-HNE show increased lipid peroxidation after MPTP and its significant reduction by RES, SDA, or PI treatment. (**E**) WB analysis of apoptosis-related proteins Bcl-2, BAX, and CC3. MPTP exposure increased pro-apoptotic BAX and CC3 and decreased anti-apoptotic Bcl-2; these changes were effectively reversed by SDA and PI administration. All protein levels were normalized to GAPDH and expressed as fold change relative to the Sham group. Data are presented as mean ± SEM (*n* = 5 per group). Statistical comparisons were performed using one-way ANOVA followed by Dunnett’s post hoc test. *, *p* < 0.05; **, *p* < 0.01; ***, *p* < 0.005; ****, *p* < 0.0001.

To determine whether the neuroprotective effects of SDA and PI were associated with improved mitochondrial homeostasis, we examined the expression of mitochondrial membrane proteins and oxidative stress markers in hippocampal lysates. WB analyses revealed a pronounced reduction in translocase of the inner membrane 23 (TIM23) and outer membrane protein TOM20 in the MPTP group, indicating mitochondrial structural impairment. Oral administration of SDA or PI markedly restored both TIM23 and TOM20 expression levels to near-control values (Figure 7A). We next evaluated mitochondrial respiratory chain complexes I–V. MPTP exposure significantly decreased the expression of NDUFB8 (complex I), SDH8 (complex II), MTCO1 (complex IV), UQCRC2 (complex III), and ATP5A (ATP Synthase Subunit Alpha; complex V), suggesting compromised oxidative phosphorylation capacity. Treatment with SDA or PI effectively normalized the abundance of all five complexes, reflecting recovery of mitochondrial bioenergetic function (Figure 7B,C). In line with these findings, the lipid peroxidation marker 4-HNE was markedly elevated in the MPTP group, consistent with enhanced oxidative damage. Both SDA and PI treatments significantly suppressed 4-HNE accumulation (Figure 7D), indicating mitigation of mitochondrial oxidative stress. Finally, we assessed apoptosis-related proteins. MPTP administration reduced the anti-apoptotic protein Bcl-2 and increased pro-apoptotic BAX and CC3. SDA and PI restored Bcl-2 expression while suppressing BAX and CC3, suggesting inhibition of mitochondrial apoptotic signaling (Figure 7E). Together, these findings support the interpretation that SDA and PI preserve mitochondrial structure, enhance respiratory chain integrity, reduce oxidative stress, and prevent apoptosis in the PD hippocampus.

## 4. Discussion

Neuroinflammation is now widely recognized as a key driver of PD pathology, contributing not only to the degeneration of dopaminergic neurons but also to the suppression of hippocampal neurogenesis and associated cognitive decline [43,44,45]. In this inflammatory network, astrocytes play a crucial role as both modulators and amplifiers of oxidative stress [46,47]. Among ROS-producing enzymes, NOX4 is particularly damaging because of its sustained ROS production [48,49], which activates downstream inflammatory mediators such as MPO and OPN. These factors create a neurotoxic environment that promotes ferroptosis and impairs regenerative processes within the hippocampal niche [27].

The current study shows that oral intake of SDA and PI, two botanically derived agents with established safety profiles, significantly reduces the NOX4–MPO–OPN pathways in the hippocampus of MPTP-induced PD mice. This suppression was accompanied by restored hippocampal neurogenesis, recovery of synaptic marker expression, and improvement of motor performance. Hippocampal neurogenesis and synaptic integrity are increasingly recognized as relevant to Parkinson’s disease beyond motor circuits, as hippocampal dysfunction has been implicated in non-motor domains such as cognition and mood [50,51]. Although non-motor outcomes were not directly assessed in the present study, the observed neurogenic and synaptic changes provide a biologically plausible link to these domains and warrant dedicated behavioral investigation in future studies.

Although both agents were administered systemically, the observed neuroprotective effects were mainly associated with astrocytic modulation. Co-localization analyses indicated that NOX4, MPO, and OPN were primarily decreased in GFAP-positive astrocytes, while neuronal NOX4 was reduced without significant changes in neuronal MPO or OPN. These results support an astrocyte-focused view of NOX4-related redox-inflammation regulation in this model. Importantly, although both agents were administered systemically, their neuroprotective effects were largely associated with astrocytic modulation. Co-localization analyses showed that SDA and PI markedly reduced NOX4, MPO, and OPN predominantly in GFAP-positive astrocytes, indicating preferential inhibition of the astrocytic redox-inflammatory pathway. While neuronal NOX4 was also decreased, the major suppression of downstream inflammatory mediators occurred in astrocytes, supporting the interpretation that these compounds primarily target an astrocyte-centered mechanism that drives neuroinflammation and hippocampal vulnerability in PD. In line with the overall attenuation of neuroinflammatory responses, hippocampal Iba-1 protein levels, a marker of microglia, were also reduced following SDA and PI treatment (Supplementary Figure S3).

Unlike synthetic drugs that usually target just a single pathway, natural products often have multiple effects, impacting oxidative stress, inflammation, neurotrophic signaling, and synaptic health all at once [52,53,54]. SDA and PI demonstrate this property. While SDA has primarily been studied for its ability to influence the hypothalamic–pituitary–adrenal axis and enhance resilience to stress-related disorders [55,56,57], PI has been linked to increased hippocampal neurogenesis and improved cognition in sleep-deprivation models [34]. These distinct biological actions may, in part, reflect their differing phytochemical profiles. SDA is enriched in carotenoid constituents such as crocin and crocetin, which have been associated with dopaminergic neuroprotection and motor improvement [40,41,58,59,60], whereas PI contains flavonoids such as vitexin and chrysin, which have been implicated in hippocampal neurogenesis, neuronal differentiation, and cognitive enhancement [61,62,63,64]. Our findings build on these known effects within the context of neurodegeneration, showing that both agents inhibit the glial redox-inflammatory cascade and protect mitochondrial health by reducing NOX4-derived oxidative stress, which ultimately aids in structural and functional recovery in the PD brain.

Interestingly, SDA was more effective in improving motor outcomes, while PI had stronger effects on hippocampal neurogenesis and neuronal differentiation. These distinct but complementary profiles support the idea of combination or stage-specific nutritional therapies, using SDA for motor symptom relief and PI for cognitive and regenerative improvements. Such strategies align with the multifaceted nature of PD, in which both motor and non-motor symptoms require coordinated treatment. Although our data suggest complementary actions of SDA and PI—SDA mainly improving motor performance and PI more effectively promoting hippocampal neurogenesis—we did not include a combined SDA+PI treatment group in this initial study. Synergistic or additive effects cannot be assumed a priori, and simultaneous administration might also mask or even oppose the specific benefits of each extract. We therefore focused on clearly defining the individual pharmacological profiles of SDA and PI while keeping animal use within IACUC-recommended limits. Future studies will be necessary to test SDA–PI combination regimens and determine whether true additive or synergistic effects occur.

From a translational perspective, repositioning SDA and PI as functional food ingredients for PD therapy offers several benefits. Both agents are standardized and already approved for human use, with documented oral bioavailability and effects on the CNS [65,66,67], which could speed up their path to clinical application. Additionally, their established safety profiles support the feasibility of long-term dietary intervention, an important factor for managing chronic neurodegenerative diseases. Although the specific molecular targets beyond the NOX4–MPO–OPN axis are not fully understood, the multi-target effects of these botanical extracts may help address the complex and overlapping pathogenic mechanisms of PD. Taken together, these findings provide preclinical evidence that SDA and PI modulate redox-inflammatory signaling and are associated with improved neurogenic and behavioral outcomes in an acute MPTP model. However, given the reliance on a single, acute toxin-based model, additional validation in chronic and genetic PD models, together with pharmacokinetic and brain exposure studies, will be necessary to establish translational relevance.

We recognize some limitations. First, although the MPTP paradigm is a widely used murine model that reliably induces dopaminergic neuronal loss and glial activation, it constitutes an acute toxin-driven insult and therefore does not recapitulate the chronic, progressive, and multifactorial features of idiopathic PD. This limitation should be considered when interpreting the translational relevance of the present findings, particularly with related to long-term disease progression and non-motor domains that are not sufficiently modeled by MPTP. Second, we employed a single 50 mg/kg dose for each extract, and future work should examine full dose–response relationships to determine minimal effective and maximal tolerated doses, and optimal therapeutic ranges. Third, Future studies should incorporate chronic or genetic PD models alongside formal pharmacokinetic evaluations to more accurately define the long-term efficacy and translational applicability of these botanical agents. Nonetheless, it provided a controlled platform for testing the glial-targeted hypothesis and clearly demonstrated histological, molecular, and behavioral benefits. The fourth, although our IF data strongly suggest astrocyte involvement, definitive proof of cell-type specificity will require conditional NOX4 knockdown in astrocytes or lineage-tracing studies. Lastly, pharmacokinetic and CNS distribution studies for SDA and PI were not performed here, although prior research confirms their ability to cross the blood–brain barrier and act within the CNS.

In summary, this study highlights SDA and PI as promising glial-targeted natural therapies that inhibit astrocytic NOX4-driven neuroinflammation and promote hippocampal neurogenesis in PD. By combining antioxidant, anti-inflammatory, neurotrophic, and synaptic-restorative properties within a safe and well-characterized botanical framework, SDA and PI offer a compelling, clinically relevant approach to disease-relevant conditions in PD and potentially other neurodegenerative disorders.

## 5. Conclusions

This study supports the interpretation that oral administration of SDA and PI provides strong neuroprotective and neuroregenerative effects in an MPTP-induced PD model by targeting astrocytic oxidative signaling. Both standardized extracts effectively inhibit the NOX4–MPO–OPN axis, a major redox-inflammatory pathway mainly active in hippocampal astrocytes, thus decreasing oxidative stress and neuroinflammation. This molecular regulation consistently correlates with restored hippocampal neurogenesis, improved synaptic integrity, increased survival of dopaminergic neurons, and notable improvements in motor performance.

The differential yet complementary actions of SDA and PI—SDA showing more potent effects on motor recovery and PI demonstrating greater enhancement of neurogenesis—highlight the potential benefit of combining these safe phytochemicals for comprehensive management of both motor and non-motor cognitive symptoms in PD. Importantly, their well-established safety and suitability for long-term use offer a clear and valuable translational advantage for future clinical development as functional foods.

Taken together, these findings highlight SDA and PI as promising glial-targeted nutritional agents capable of modifying disease progression through a multifaceted combination of antioxidant, anti-inflammatory, and neurotrophic mechanisms. Future research involving chronic or genetic PD models, astrocyte-specific NOX4 modulation, and detailed pharmacokinetic profiling will be crucial to fully validate and optimize their therapeutic potential. Overall, this work provides a strong and convincing foundation for advancing SDA and PI as safe, versatile, and clinically relevant nutritional interventions for PD and potentially other neurodegenerative disorders marked by oxidative stress and impaired neurogenesis. While not implying disease modification, our findings highlight SDA and PI as promising neuroprotective agents that target astrocytic oxidative signaling linked to Parkinson’s disease pathology.

## Data Availability

The original contributions presented in this study are included in this article. Further inquiries can be directed to the corresponding author.

## Supplementary Materials

The following supporting information can be downloaded at: https://www.mdpi.com/article/10.3390/nu18010055/s1. Figure S1: Experimental timeline for extract administration, MPTP induction, behavioral testing, and tissue collection. Figure S2: Longitudinal changes in body weight, food intake, and water consumption during the 5-week experimental period. Figure S3. Natural extract treatments attenuate, but differentially modulate, hippocampal microglial activation in MPTP-induced Parkinson’s mice.

## Abbreviations

4-HNE	4-Hydroxynonenal, a marker of lipid peroxidation
ATP5A	ATP Synthase Subunit Alpha
BAX	Bcl-2-Associated X Protein
Bcl-2	B-Cell Lymphoma 2
BrdU	5-Bromo-2′-Deoxyuridine
CA1/CA2/CA3	Cornu Ammonis Fields 1–3 of the hippocampus
CC3	Cleaved Caspase-3
DCX	Doublecortin
D.W.	Distilled Water
GAPDH	Glyceraldehyde-3-Phosphate Dehydrogenase
GFAP	Glial Fibrillary Acidic Protein
GCL	Granule Cell Layer
HRP	Horseradish peroxidase
Iba-1	Ionized calcium-binding adaptor molecule 1
IHC	Immunohistochemistry
IF	Immunofluorescence
ML	Molecular Layer
MPO	Myeloperoxidase
MPTP	1-Methyl-4-Phenyl-1,2,3,6-Tetrahydropyridine
NeuN	Neuronal Nuclei
NOX4	NADPH Oxidase 4
OPN	Osteopontin
OXPHOS	Oxidative Phosphorylation Complexes I–V
PFA	paraformaldehyde
PD	Parkinson’s Disease
PI	*Passiflora incarnata* L.
PSD95	Postsynaptic Density Protein-95
RES	Resveratrol
ROS	Reactive Oxygen Species
SDA	Saffron-Derived Antioxidant (Crocus sativus Extract)
SEM	Standard Error of the Mean
SGZ	Subgranular Zone
SNc	Substantia Nigra Pars Compacta
SO	Stratum Oriens
SP	Stratum Pyramidale
SR	Stratum Radiatum
TH	Tyrosine Hydroxylase
TIM23	Translocase of the Inner Mitochondrial Membrane-23
TOM20	Translocase of the Outer Mitochondrial Membrane-20
WB	Western Blot

## References

[1] Li M., Chen M., Li H., Gao D., Zhao L., Zhu M. (2024). Glial cells improve Parkinson’s disease by modulating neuronal function and regulating neuronal ferroptosis. Front. Cell Dev. Biol..

[2] Wang N., Xiao X., Chen Z., Xu K., Cao X., Kou D., Zeng J. (2025). Glial Cell Crosstalk in Parkinson’s Disease: Mechanisms, Implications, and Therapeutic Strategies. Fundam. Res..

[3] Lim D., Matute C., Cavaliere F., Verkhratsky A. (2025). Neuroglia in neurodegeneration: Alzheimer, Parkinson, and Huntington disease. Handb. Clin. Neurol..

[4] Zhou Z.D., Yi L.X., Wang D.Q., Lim T.M., Tan E.K. (2023). Role of dopamine in the pathophysiology of Parkinson’s disease. Transl. Neurodegener..

[5] Zhang Y.M., Qi Y.B., Gao Y.N., Chen W.G., Zhou T., Zang Y., Li J. (2023). Astrocyte metabolism and signaling pathways in the CNS. Front. Neurosci..

[6] Fisher T.M., Liddelow S.A. (2024). Emerging roles of astrocytes as immune effectors in the central nervous system. Trends Immunol..

[7] Linnerbauer M., Wheeler M.A., Quintana F.J. (2020). Astrocyte Crosstalk in CNS Inflammation. Neuron.

[8] Dauer W., Przedborski S. (2003). Parkinson’s disease: Mechanisms and models. Neuron.

[9] McKinnon C., De Snoo M.L., Gondard E., Neudorfer C., Chau H., Ngana S.G., O’Hara D.M., Brotchie J.M., Koprich J.B., Lozano A.M. (2020). Early-onset impairment of the ubiquitin-proteasome system in dopaminergic neurons caused by α-synuclein. Acta Neuropathol. Commun..

[10] Yadav D., Kumar P. (2022). Restoration and targeting of aberrant neurotransmitters in Parkinson’s disease therapeutics. Neurochem. Int..

[11] Koszla O., Solek P. (2024). Misfolding and aggregation in neurodegenerative diseases: Protein quality control machinery as potential therapeutic clearance pathways. Cell Commun. Signal..

[12] Kamila P., Kar K., Chowdhury S., Chakraborty P., Dutta R., Singh A., Prajapati B.G. (2025). Effect of neuroinflammation on the progression of Alzheimer’s disease and its significant ramifications for novel anti-inflammatory treatments. IBRO Neurosci. Rep..

[13] Gudkov S.V., Burmistrov D.E., Kondakova E.V., Sarimov R.M., Yarkov R.S., Franceschi C., Vedunova M.V. (2023). An emerging role of astrocytes in aging/neuroinflammation and gut-brain axis with consequences on sleep and sleep disorders. Ageing Res. Rev..

[14] Kwon H.S., Koh S.H. (2020). Neuroinflammation in neurodegenerative disorders: The roles of microglia and astrocytes. Transl. Neurodegener..

[15] Leal-Galicia P., Chavez-Hernandez M.E., Mata F., Mata-Luevanos J., Rodriguez-Serrano L.M., Tapia-de-Jesus A., Buenrostro-Jauregui M.H. (2021). Adult Neurogenesis: A Story Ranging from Controversial New Neurogenic Areas and Human Adult Neurogenesis to Molecular Regulation. Int. J. Mol. Sci..

[16] Snapyan M., Desmeules F., Munro J., Berard M., Saikali S., Gould P.V., Richer M., Pourcher E., Langlois M., Dufresne A.M. (2025). Adult Neurogenesis in the Subventricular Zone of Patients with Huntington’s and Parkinson’s Diseases and following Long-Term Treatment with Deep Brain Stimulation. Ann. Neurol..

[17] Kaneko N., Sawamoto K. (2009). Adult neurogenesis and its alteration under pathological conditions. Neurosci. Res..

[18] Anacker C., Hen R. (2017). Adult hippocampal neurogenesis and cognitive flexibility-linking memory and mood. Nat. Rev. Neurosci..

[19] Sun D., Mei L., Xiong W.C. (2023). Dorsal Dentate Gyrus, a Key Regulator for Mood and Psychiatric Disorders. Biol. Psychiatry.

[20] Mandyam C.D., Koob G.F. (2012). The addicted brain craves new neurons: Putative role for adult-born progenitors in promoting recovery. Trends Neurosci..

[21] Wang Y., Xia Y., Kou L., Yin S., Chi X., Li J., Sun Y., Wu J., Zhou Q., Zou W. (2023). Astrocyte-to-neuron reprogramming and crosstalk in the treatment of Parkinson’s disease. Neurobiol. Dis..

[22] Booth H.D.E., Hirst W.D., Wade-Martins R. (2017). The Role of Astrocyte Dysfunction in Parkinson’s Disease Pathogenesis. Trends Neurosci..

[23] Escartin C., Galea E., Lakatos A., O’Callaghan J.P., Petzold G.C., Serrano-Pozo A., Steinhauser C., Volterra A., Carmignoto G., Agarwal A. (2021). Reactive astrocyte nomenclature, definitions, and future directions. Nat. Neurosci..

[24] Valori C.F., Guidotti G., Brambilla L., Rossi D. (2019). Astrocytes: Emerging Therapeutic Targets in Neurological Disorders. Trends Mol. Med..

[25] Chang K.H., Chen C.M. (2020). The Role of Oxidative Stress in Parkinson’s Disease. Antioxidants.

[26] He J., Zhu G., Wang G., Zhang F. (2020). Oxidative Stress and Neuroinflammation Potentiate Each Other to Promote Progression of Dopamine Neurodegeneration. Oxidative Med. Cell. Longev..

[27] Boonpraman N., Yoon S., Kim C.Y., Moon J.S., Yi S.S. (2023). NOX4 as a critical effector mediating neuroinflammatory cytokines, myeloperoxidase and osteopontin, specifically in astrocytes in the hippocampus in Parkinson’s disease. Redox Biol..

[28] Boonpraman N., Yi S.S. (2024). NADPH oxidase 4 (NOX4) as a biomarker and therapeutic target in neurodegenerative diseases. Neural Regen. Res..

[29] Park M.W., Cha H.W., Kim J., Kim J.H., Yang H., Yoon S., Boonpraman N., Yi S.S., Yoo I.D., Moon J.S. (2021). NOX4 promotes ferroptosis of astrocytes by oxidative stress-induced lipid peroxidation via the impairment of mitochondrial metabolism in Alzheimer’s diseases. Redox Biol..

[30] Obeso I., Wilkinson L., Casabona E., Bringas M.L., Alvarez M., Alvarez L., Pavon N., Rodriguez-Oroz M.C., Macias R., Obeso J.A. (2011). Deficits in inhibitory control and conflict resolution on cognitive and motor tasks in Parkinson’s disease. Exp. Brain Res..

[31] Devos D., Moreau C., Dujardin K., Cabantchik I., Defebvre L., Bordet R. (2013). New pharmacological options for treating advanced Parkinson’s disease. Clin. Ther..

[32] Riederer P., Strobel S., Nagatsu T., Watanabe H., Chen X., Loschmann P.A., Sian-Hulsmann J., Jost W.H., Muller T., Dijkstra J.M. (2025). Levodopa treatment: Impacts and mechanisms throughout Parkinson’s disease progression. J. Neural Transm..

[33] Kim C.Y., Ko K., Choi S.H., Jo M., Kim J., Yoon S., Yi I.J., Moran-Valero M.I., Kwon M.Y., Sohn J. (2023). Effects of Saffron Extract (Affron^®^) with 100 mg/kg and 200 mg/kg on Hypothalamic-Pituitary-Adrenal Axis and Stress Resilience in Chronic Mild Stress-Induced Depression in Wistar Rats. Nutrients.

[34] Kim G.H., Lim K., Yang H.S., Lee J.K., Kim Y., Park S.K., Kim S.H., Park S., Kim T.H., Moon J.S. (2019). Improvement in neurogenesis and memory function by administration of Passiflora incarnata L. extract applied to sleep disorder in rodent models. J. Chem. Neuroanat..

[35] Anandhan A., Tamilselvam K., Vijayraja D., Ashokkumar N., Rajasankar S., Manivasagam T. (2010). Resveratrol attenuates oxidative stress and improves behaviour in 1 -methyl-4-phenyl-1,2,3,6-tetrahydropyridine (MPTP) challenged mice. Ann. Neurosci..

[36] Blanchet J., Longpre F., Bureau G., Morissette M., DiPaolo T., Bronchti G., Martinoli M.G. (2008). Resveratrol, a red wine polyphenol, protects dopaminergic neurons in MPTP-treated mice. Prog. Neuropsychopharmacol. Biol. Psychiatry.

[37] Lofrumento D.D., Nicolardi G., Cianciulli A., De Nuccio F., La Pesa V., Carofiglio V., Dragone T., Calvello R., Panaro M.A. (2014). Neuroprotective effects of resveratrol in an MPTP mouse model of Parkinson’s-like disease: Possible role of SOCS-1 in reducing pro-inflammatory responses. Innate Immun..

[38] Guo Y.J., Dong S.Y., Cui X.X., Feng Y., Liu T., Yin M., Kuo S.H., Tan E.K., Zhao W.J., Wu Y.C. (2016). Resveratrol alleviates MPTP-induced motor impairments and pathological changes by autophagic degradation of alpha-synuclein via SIRT1-deacetylated LC3. Mol. Nutr. Food Res..

[39] Lu K.T., Ko M.C., Chen B.Y., Huang J.C., Hsieh C.W., Lee M.C., Chiou R.Y., Wung B.S., Peng C.H., Yang Y.L. (2008). Neuroprotective effects of resveratrol on MPTP-induced neuron loss mediated by free radical scavenging. J. Agric. Food Chem..

[40] Chrastina M., Drafi F., Pruzinska K., Ponist S., Kamga K.S., Khademnematolahi S., Bilka F., Novak P., Paskova L., Bauerova K. (2023). Crocus sativus L. Extract (Saffron) Effectively Reduces Arthritic and Inflammatory Parameters in Monotherapy and in Combination with Methotrexate in Adjuvant Arthritis. Nutrients.

[41] Dong N., Dong Z., Chen Y., Gu X. (2020). Crocetin Alleviates Inflammation in MPTP-Induced Parkinson’s Disease Models through Improving Mitochondrial Functions. Parkinson’s Dis..

[42] Tamegart L., Abbaoui A., Makbal R., Zroudi M., Bouizgarne B., Bouyatas M.M., Gamrani H. (2019). Crocus sativus restores dopaminergic and noradrenergic damages induced by lead in Meriones shawi: A possible link with Parkinson’s disease. Acta Histochem..

[43] Wu A., Zhang J. (2023). Neuroinflammation, memory, and depression: New approaches to hippocampal neurogenesis. J. Neuroinflamm..

[44] Hirsch E.C., Hunot S. (2009). Neuroinflammation in Parkinson’s disease: A target for neuroprotection?. Lancet Neurol..

[45] Ryan S.M., Nolan Y.M. (2016). Neuroinflammation negatively affects adult hippocampal neurogenesis and cognition: Can exercise compensate?. Neurosci. Biobehav. Rev..

[46] Chen Y., Qin C., Huang J., Tang X., Liu C., Huang K., Xu J., Guo G., Tong A., Zhou L. (2020). The role of astrocytes in oxidative stress of central nervous system: A mixed blessing. Cell Prolif..

[47] Won W., Bhalla M., Lee J.H., Lee C.J. (2025). Astrocytes as Key Regulators of Neural Signaling in Health and Disease. Annu. Rev. Neurosci..

[48] Weyemi U., Dupuy C. (2012). The emerging role of ROS-generating NADPH oxidase NOX4 in DNA-damage responses. Mutat. Res..

[49] Canugovi C., Stevenson M.D., Vendrov A.E., Hayami T., Robidoux J., Xiao H., Zhang Y.Y., Eitzman D.T., Runge M.S., Madamanchi N.R. (2019). Increased mitochondrial NADPH oxidase 4 (NOX4) expression in aging is a causative factor in aortic stiffening. Redox Biol..

[50] Kohl Z., Ben Abdallah N., Vogelgsang J., Tischer L., Deusser J., Amato D., Anderson S., Muller C.P., Riess O., Masliah E. (2016). Severely impaired hippocampal neurogenesis associates with an early serotonergic deficit in a BAC alpha-synuclein transgenic rat model of Parkinson’s disease. Neurobiol. Dis..

[51] Belloso-Iguerategui A., Zamarbide M., Merino-Galan L., Rodriguez-Chinchilla T., Gago B., Santamaria E., Fernandez-Irigoyen J., Cotman C.W., Prieto G.A., Quiroga-Varela A. (2023). Hippocampal synaptic failure is an early event in experimental parkinsonism with subtle cognitive deficit. Brain.

[52] Rahman M.M., Wang X., Islam M.R., Akash S., Supti F.A., Mitu M.I., Harun-Or-Rashid M., Aktar M.N., Khatun Kali M.S., Jahan F.I. (2022). Multifunctional role of natural products for the treatment of Parkinson’s disease: At a glance. Front. Pharmacol..

[53] Nahar L., Charoensup R., Kalieva K., Habibi E., Guo M., Wang D., Kvasnica M., Onder A., Sarker S.D. (2025). Natural products in neurodegenerative diseases: Recent advances and future outlook. Front. Pharmacol..

[54] Mohd Sairazi N.S., Sirajudeen K.N.S. (2020). Natural Products and Their Bioactive Compounds: Neuroprotective Potentials against Neurodegenerative Diseases. Evid. Based Complement. Alternat. Med..

[55] Abbaszade-Cheragheali A., Beheshti F., Kakhki S., Khatibi S.R., Dehnokhalaji F., Akbari E., Fathi H., Safari Farimani S. (2022). Crocin, the main active saffron (*Crocus sativus* L.) constituent, as a potential candidate to prevent anxiety and depressive-like behaviors induced by unpredictable chronic mild stress. Neurosci. Lett..

[56] Siddiqui S.A., Ali Redha A., Snoeck E.R., Singh S., Simal-Gandara J., Ibrahim S.A., Jafari S.M. (2022). Anti-Depressant Properties of Crocin Molecules in Saffron. Molecules.

[57] Christodoulou E., Kadoglou N.P., Kostomitsopoulos N., Valsami G. (2015). Saffron: A natural product with potential pharmaceutical applications. J. Pharm. Pharmacol..

[58] Ben El Caid M., Ait Haddou M., El Asri O., Aboudlou L., Atyane L.H., Ramteke V., Ait Hammou R. (2025). Neuroprotective potential of saffron metabolites in Parkinson’s disease. PharmaNutrition.

[59] Bian Y., Zhao C., Lee S.M. (2020). Neuroprotective Potency of Saffron Against Neuropsychiatric Diseases, Neurodegenerative Diseases, and Other Brain Disorders: From Bench to Bedside. Front. Pharmacol..

[60] Inoue E., Suzuki T., Shimizu Y., Sudo K., Kawasaki H., Ishida N. (2021). Saffron ameliorated motor symptoms, short life span and retinal degeneration in Parkinson’s disease fly models. Gene.

[61] Dehghani M.A., Meftahi G.H., Khorasgani E.M. (2025). Passiflora incarnate extract attenuates neuronal loss and memory impairment in stressed rats. Brain Res..

[62] Hu M., Li F., Wang W. (2018). Vitexin protects dopaminergic neurons in MPTP-induced Parkinson’s disease through PI3K/Akt signaling pathway. Drug Des. Devel. Ther..

[63] Jawna-Zboinska K., Blecharz-Klin K., Joniec-Maciejak I., Wawer A., Pyrzanowska J., Piechal A., Mirowska-Guzel D., Widy-Tyszkiewicz E. (2016). *Passiflora incarnata* L. Improves Spatial Memory, Reduces Stress, and Affects Neurotransmission in Rats. Phytother. Res..

[64] Lima L.K.F., Pereira S.K.S., Junior R., Santos F., Nascimento A.S., Feitosa C.M., Figueredo J.S., Cavalcante A.D.N., Araujo E., Rai M. (2018). A Brief Review on the Neuroprotective Mechanisms of Vitexin. BioMed Res. Int..

[65] Omidkhoda S.F., Hosseinzadeh H. (2022). Saffron and its active ingredients against human disorders: A literature review on existing clinical evidence. Iran. J. Basic Med. Sci..

[66] Bej E., Volpe A.R., Cesare P., Cimini A., d’Angelo M., Castelli V. (2024). Therapeutic potential of saffron in brain disorders: From bench to bedside. Phytother. Res..

[67] da Fonseca L.R., Rodrigues R.A., Ramos A.S., da Cruz J.D., Ferreira J.L.P., Silva J.R.A., Amaral A.C.F. (2020). Herbal Medicinal Products from Passiflora for Anxiety: An Unexploited Potential. Sci. World J..

