# Supplementary figures and images for: Oral Administration of Astrocyte-Targeted Natural Antioxidants Suppress NOX4-Driven Neuroinflammation and Restore Hippocampal Neurogenesis in MPTP-Induced Parkinson’s Disease Mouse Model
Source: Nutrients. 2025 Dec 23;18(1):55. doi: 10.3390/nu18010055 (PMC12787697; doi:10.3390/nu18010055)

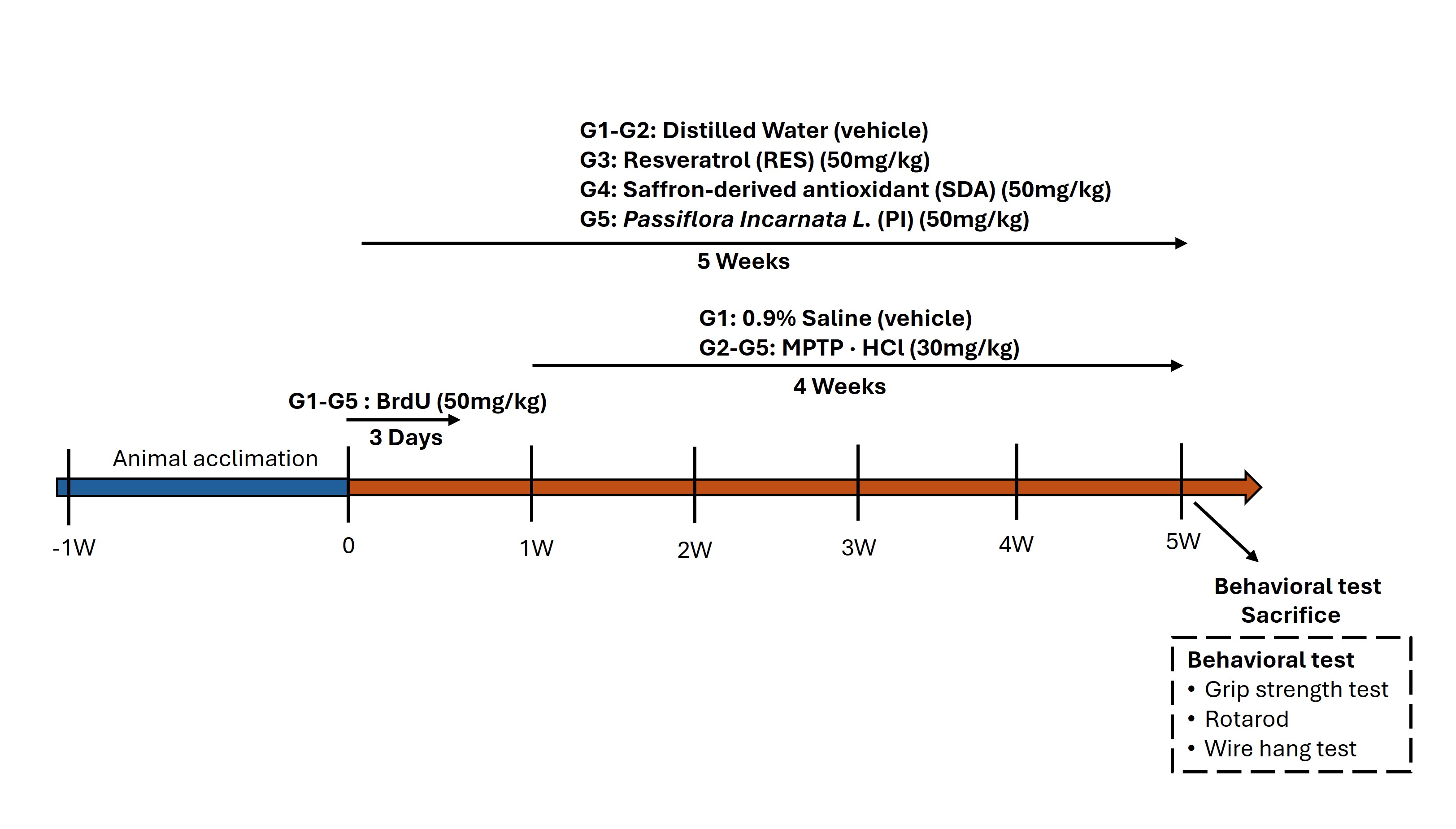

Supplement: Supplementary file 1 [file nutrients-18-00055-s001.zip › Figure S1.jpg]

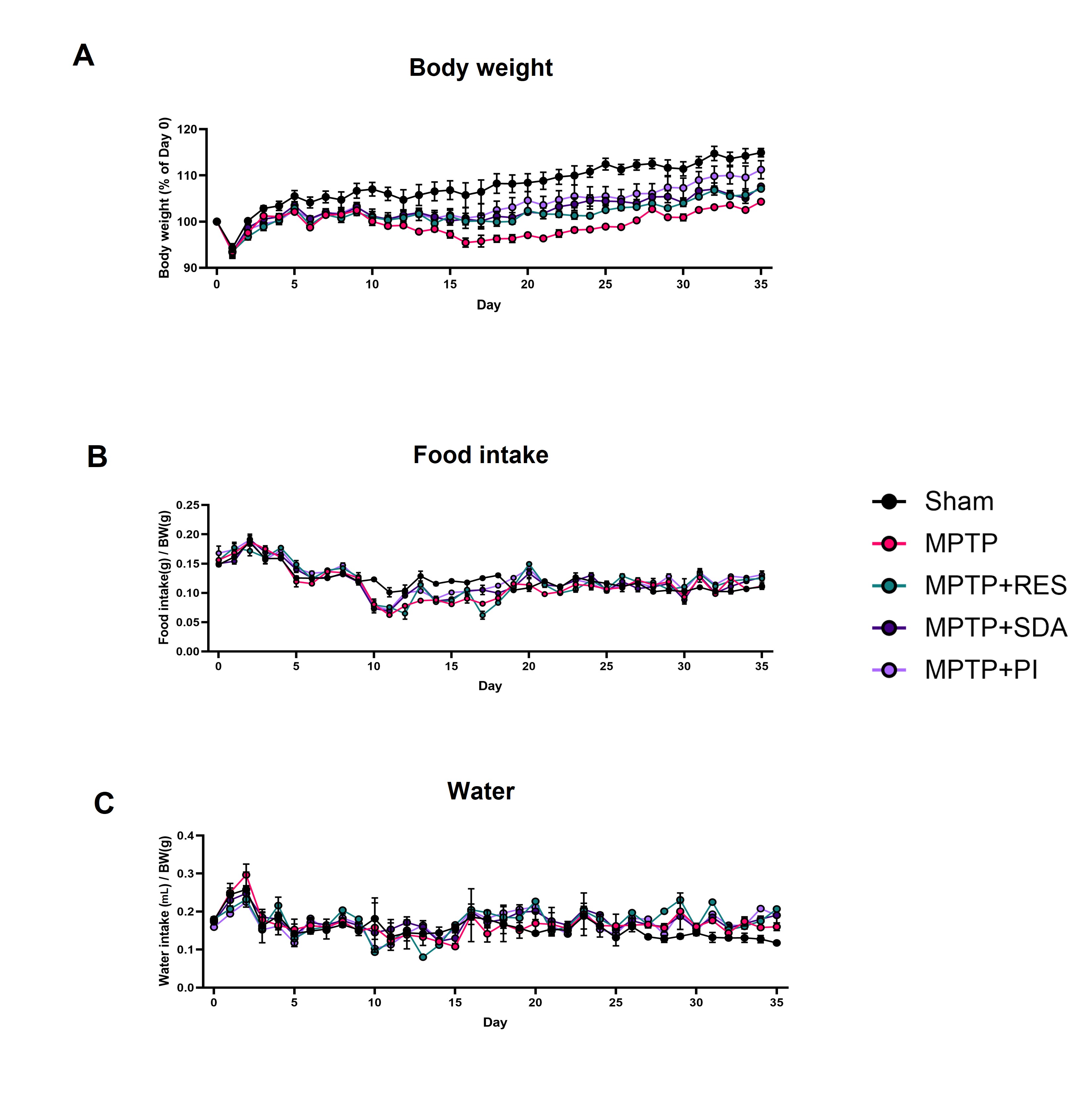

Supplement: Supplementary file 1 [file nutrients-18-00055-s001.zip › Figure S2.jpg]

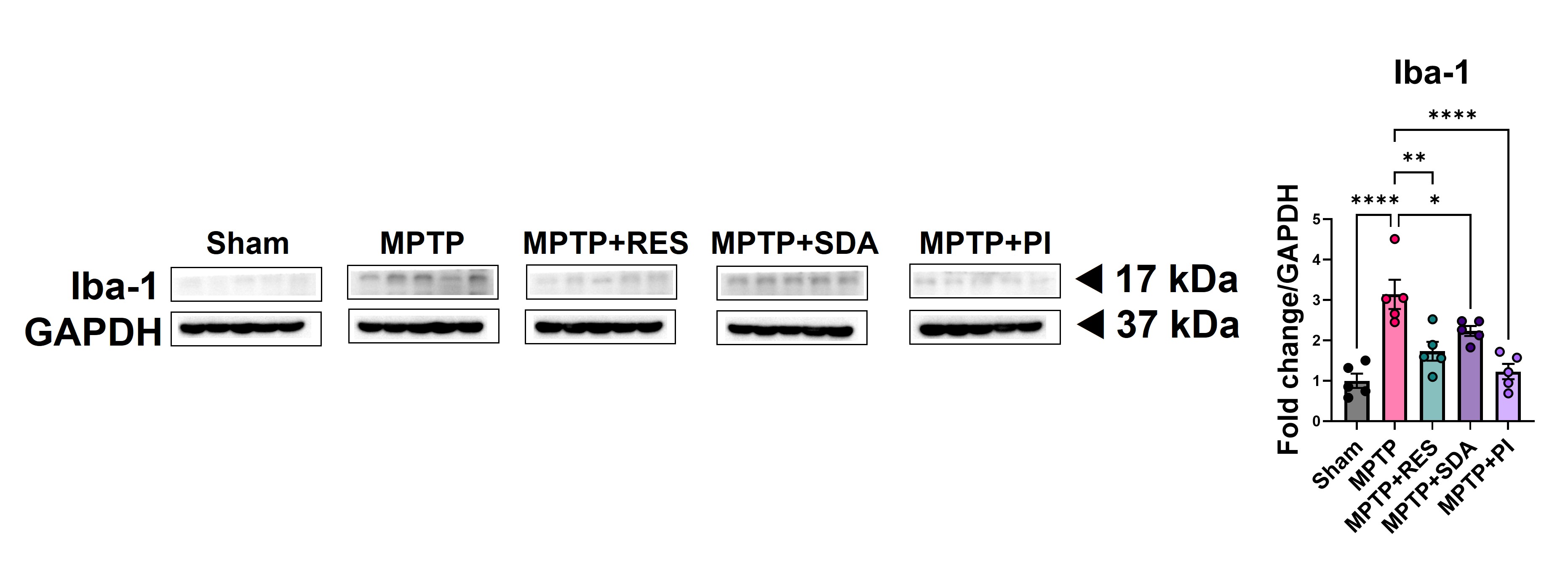

Supplement: Supplementary file 1 [file nutrients-18-00055-s001.zip › Figure S3.jpg]
